# Experiences of NICU Nurses Facing Perinatal Death: A Phenomenological Study

**DOI:** 10.3390/children13060795

**Published:** 2026-06-09

**Authors:** Sara González-Astray, Cristo-Manuel Marrero-González, Irene González-Pérez, Judith Arbelo-Molina, Alfonso Miguel García Hernández, Aythamy González-Darias

**Affiliations:** 1 Unidad de Cuidados Intensivos Neonatales, Complejo Hospitalario Universitario de Canarias (CHUC), 38320 Santa Cruz de Tenerife, Spain; gonzalezastraysara@gmail.com (S.G.-A.); jarbmol@gobiernodecanarias.org (J.A.-M.); agonzada@ull.edu.es (A.G.-D.); 2 Facultad de Enfermería, Departamento de Enfermería, Universidad de La Laguna, 38200 Santa Cruz de Tenerife, Spain; almigar@ull.edu.es

**Keywords:** perinatal death, neonatal intensive care, NICU nurses, bereavement, neonatal palliative care, compassion fatigue, moral distress

## Abstract

**Highlights:**

**What are the main findings?**

- NICU nurses experience perinatal death as a profound emotional and ethical challenge, marked by grief, guilt, moral distress, and strong therapeutic bonds.
- Peer support is a key coping resource, while insufficient institutional support, limited training, and inadequate infrastructure hinder humanized end-of-life care.

**What are the implications of the main findings?**

- Strengthening institutional strategies—including structured debriefing, psychological support, and training in neonatal palliative care—may be important for nurses’ well-being and practice.
- Improving care environments and integrating humanization into NICU practice may contribute to more compassionate, sustainable, and ethically grounded care.

**Abstract:**

**Background:** Perinatal death in Neonatal Intensive Care Units (NICUs) represents one of the most emotionally challenging experiences for nurses. While parental bereavement has been widely studied, nurses’ experiences in neonatal end-of-life care remain insufficiently explored. **Objective:** To explore the experiences of NICU nurses facing perinatal death, focusing on emotional, professional, and institutional dimensions of care. **Methods:** A qualitative study with a hermeneutic phenomenological approach was conducted through ten semi-structured interviews with NICU nurses in a tertiary-level hospital in Spain. Data were analyzed using reflexive thematic analysis supported by NVivo 15. **Results:** Seven main themes were identified: emotional responses, therapeutic bond, coping strategies, perceived institutional support, training needs, infrastructure and humanization, and professional repercussions. Nurses reported intense emotional responses, including sadness, guilt, moral distress, and perceived failure, particularly in cases of prolonged hospitalization or unexpected death. Peer support emerged as a key protective factor, whereas the lack of formal psychological support and adequate infrastructural conditions were identified as significant gaps. **Conclusions:** Strengthening institutional support for NICU nurses through structured debriefing, accessible psychological services, targeted training in neonatal palliative care, and improved care environments may enhance their well-being and resilience, contributing to sustainable and compassionate clinical practice.

## 1. Introduction

Perinatal death remains a complex and emotionally demanding reality in Neonatal Intensive Care Units (NICUs). Repeated exposure to neonatal loss has been consistently associated with burnout, compassion fatigue, moral distress, and psychological vulnerability among pediatric and neonatal nurses [1,2,3,4].

The process surrounding patient death is inherently complex and challenging for healthcare professionals, particularly when they feel insufficiently prepared to manage the emotional and ethical implications for themselves. In neonatal settings, this experience becomes especially significant due to the perceived injustice and emotional intensity associated with the death of a newborn, often leading to heightened levels of anxiety, helplessness, and moral distress among nurses [5,6].

In addition, NICUs represent highly demanding environments that pose significant emotional and professional challenges, especially for less experienced nurses [7]. Evidence suggests a high prevalence of anxiety, depression, burnout, and post-traumatic stress symptoms among neonatal and pediatric healthcare professionals [3,8,9]. These conditions are often exacerbated by the ethical complexity of end-of-life decision-making, in which nurses must navigate relationships with families, interdisciplinary teams, and their own professional values, frequently experiencing moral distress [10,11].

Furthermore, previous research highlights the need for enhanced training, institutional support, and structured protocols in neonatal end-of-life care, particularly in Ibero-American contexts [12]. Nurses not only experience grief following patient loss but also require adequate preparation and psychological support to cope with the emotional burden inherent in their role [13]. Institutional environments that promote education in pediatric palliative care and provide emotional support systems have been associated with enhanced well-being and greater retention of NICU nurses [2].

In an increasingly technology-driven healthcare context, there is a growing need to balance technical care with humane and family-centered approaches that support both families and professionals. Initiatives such as peer support groups and reflective spaces have been shown to enhance self-compassion and emotional resilience among neonatal nurses [14]. In NICU settings, technological intensity is not merely a contextual feature but a central element shaping the experience of care. Continuous monitoring, invasive procedures, life-sustaining technologies, prognostic uncertainty, and rapid clinical deterioration may reinforce a care environment strongly oriented toward survival and technical intervention. When death occurs within this setting, nurses may navigate tensions between the possibilities offered by life-sustaining technologies, ethical decision-making, and the need to provide emotionally present and humanized end-of-life care [10,11].

Taken together, although previous studies have examined perinatal bereavement, grief, burnout, compassion fatigue, and moral distress among neonatal and pediatric nurses, less is known about the ways in which NICU nurses interpret and give meaning to perinatal death as a situated, lived, relational, ethical, and professional experience. In particular, the interaction between emotional responses, therapeutic bonding with neonates and families, coping strategies, perceived institutional support, and professional repercussions remains insufficiently understood from an integrated phenomenological perspective. This gap is especially relevant in Spanish and Ibero-American neonatal care contexts, where previous research has highlighted the need for more structured training, institutional support, and humanized approaches to neonatal end-of-life care [12].

These dimensions were prioritized because perinatal death in NICU settings involves not only emotional suffering but also ethical uncertainty, relational proximity, professional self-questioning, and organizational conditions that shape how nurses cope with and respond to loss. A hermeneutic phenomenological approach is therefore especially appropriate to explore how nurses make sense of these experiences within their clinical, relational, and institutional contexts [15,16].

Therefore, the aim of this study was to explore the lived experiences of NICU nurses facing perinatal death, with particular attention to how emotional, ethical, relational, professional, and institutional dimensions shape the meaning of neonatal end-of-life care.

## 2. Materials and Methods

### 2.1. Design

A qualitative study with a hermeneutic phenomenological design was conducted using semi-structured individual interviews. This approach was selected to explore nurses’ experiences of perinatal death in NICU settings.

The study was reported following the *Consolidated Criteria for Reporting Qualitative Research* (COREQ) guidelines [17], using the Spanish-adapted version NVivo 15 proposed by Quemba-Mesa et al. [18].

### 2.2. Theoretical Framework

This study was grounded in Heideggerian hermeneutic phenomenology, which focuses on understanding human experience as situated, relational, and interpretative. Within this perspective, individuals are understood as *being-in-the-world* (*Dasein*), where meaning emerges through lived experience rather than abstract description. In nursing research, this framework allows for the exploration of care as an existential and relational phenomenon, extending beyond technical practice to include emotional, ethical, and contextual dimensions. From this perspective, caring is not merely an instrumental activity but a meaningful engagement with others, particularly in situations involving vulnerability, finitude, and end-of-life care. This approach is especially relevant for examining neonatal nurses’ experiences of perinatal death, as it enables an in-depth understanding of how professionals interpret and make sense of care, loss, and professional identity within their clinical practice [15,16].

### 2.3. Study Setting and Recruitment

This study was conducted in the Neonatal Intensive Care Unit (NICU) of the Complejo Hospitalario Universitario de Canarias, a tertiary care hospital of the Canary Islands Health Service in Tenerife, Spain. The Canary Islands have a population of 2,213,016 inhabitants, with 14.2% of foreign nationality, and are served by a public healthcare network comprising three tertiary and five regional hospitals. The Hospital Universitario de Canarias reported 1704 births in 2024, including 1640 singletons, 26 twins, and 1 triplet birth, with 9.4% of newborns born preterm (22–36 weeks). In 2025, the NICU recorded 5 neonatal deaths, reflecting low mortality but high emotional and professional impact on the nursing and healthcare team. This unit employs approximately 48 nurses and provides specialized neonatal care for a large regional population.

Data were collected during the last quarter of 2025, and analysis was conducted in the first quarter of 2026. Participants were recruited during an informational meeting with NICU nursing staff, at which the principal investigator explained the study aims, procedures, voluntary nature of participation, and confidentiality safeguards. All eligible nurses working in the NICU at the time of recruitment were informed about the study. Nurses who expressed willingness to participate contacted the research team or confirmed their interest after the meeting.

Although maximum variation sampling was not formally used, purposive sampling sought to include NICU nurses with different experiential backgrounds. Purposive sampling was informed by the aim of capturing experiential diversity among NICU nurses who had cared for newborns and families in situations of perinatal death. Variation in years of NICU experience, gender, parental status, and previous palliative-care training were considered analytically relevant, as these characteristics could influence how nurses experienced, interpreted, and coped with neonatal loss. Rather than seeking statistical representativeness, the study aimed to capture a range of perspectives, allowing for a more comprehensive understanding of the phenomenon within the specific clinical context.

Sampling continued until theoretical saturation was reached. Saturation was assessed iteratively during data collection and preliminary analysis. After each interview, the research team reviewed emerging codes, recurrent experiential meanings, and potential themes. Saturation was considered achieved when the final interviews no longer generated new relevant codes, experiential meanings, or thematic insights and when the research team reached analytic consensus that the existing data provided sufficient depth and variation to address the study aim.

A total of ten nurses (*n* = 10) were included in the final sample. The exact number of nurses who declined participation or did not respond was not formally recorded. Therefore, the possibility of self-selection bias cannot be excluded, as nurses with greater interest in bereavement care or stronger emotional experiences may have been more likely to participate.

All participants had previous clinical experience caring for newborns and families in situations of perinatal death. Sociodemographic and professional characteristics, including age, gender, parenthood status, years of NICU experience, and previous training in palliative, bereavement, or end-of-life care, are presented in Table 1.

### 2.4. Inclusion and Exclusion Criteria

Inclusion criteria required participants to have at least one year of professional experience in the NICU and to be actively working at the time of the interview, either full-time or with a workload reduction of up to 50%. All participants provided informed consent prior to participation.

Exclusion criteria included being on leave (e.g., maternity, medical, or personal leave) at the time of data collection or having less than one year of professional experience in the NICU.

### 2.5. Data Collection

Data were collected through semi-structured individual interviews based on a predefined interview guide. This approach allowed participants to freely express their experiences while ensuring consistency across interviews. Interviews were conducted in a private room in the hospital to ensure confidentiality and a comfortable environment for participants.

The interviews were conducted by two members of the research team, A.G.D. and S.G.A., both of whom were familiar with the NICU context. A.G.D. had previously worked in the unit, and S.G.A. was undertaking post-registration specialization in Pediatric Nursing in the same clinical setting. This familiarity facilitated contextual understanding and sensitivity towards the phenomenon under study. However, given the potential influence of professional proximity on participants’ narratives, several measures were adopted to support methodological rigor.

Prior to each interview, participants were informed of the voluntary nature of participation, their right to decline to respond to any question, and the measures in place to ensure confidentiality and anonymity. The interviewers adopted a respectful, non-directive, and empathetic approach, allowing participants to narrate their experiences in their own terms. The semi-structured guide was used flexibly, and additional probing questions were introduced when necessary to deepen participants’ narratives. Examples of probes included: “Could you tell me more about that experience?”, “How did that situation affect you emotionally or professionally?”, “What kind of support would have been helpful at that moment?”, and “How did the relationship with the family influence the way you experienced that situation?”

All interviews were audio-recorded using a digital recording device and stored in MP3 format. Audio recordings were transcribed verbatim using dedicated transcription software and subsequently reviewed by the research team to ensure accuracy and completeness. Each interview lasted approximately 30 min. The interview guide is presented in Table 2 and was developed in alignment with the study aims and research design.

### 2.6. Data Analysis

Data were analyzed using reflexive thematic analysis as described by Braun & Clarke [19], supported by NVivo 15. This analytic approach was selected due to its epistemological compatibility with hermeneutic phenomenology, which conceptualizes meaning as contextual, relational, and interpretative. The use of reflexive thematic analysis was considered epistemologically coherent with Heideggerian hermeneutic phenomenology. Both approaches are grounded in an interpretative understanding of meaning, recognizing that experiences are situated, relational, and shaped by context. In this study, reflexive thematic analysis was not applied as a purely descriptive or positivist coding technique but as a flexible and recursive analytic process for identifying patterns of meaning within participants’ narratives. Hermeneutic phenomenology guided the interpretation of nurses’ lived experiences, while reflexive thematic analysis provided a systematic procedure for organizing codes, developing themes, and constructing an analytic narrative grounded in the data. Interviews were conducted and analyzed in Spanish. Codes and themes were progressively developed and labeled in English to facilitate reporting. Quotations were translated into English for publication purposes.

Analysis followed six recursive phases: familiarization with the data through repeated reading of transcripts; generation of initial codes focused on experiential aspects of neonatal end-of-life care; development of themes through the clustering of related codes; review and refinement of themes in relation to both coded data and the full dataset; definition and naming of themes; and production of the final analytic narrative.

Initial coding was conducted through repeated reading of the transcripts and identification of meaningful units related to nurses’ experiences of perinatal death. Codes were generated inductively and progressively refined through an iterative process of comparison across transcripts. The research team reviewed the emerging codes collaboratively, discussing their interpretative meaning and their relationship with the broader experiential context. Disagreements were addressed through reflexive discussion and consensus, rather than through calculation of inter-coder agreement, in line with the principles of reflexive thematic analysis.

Codes were subsequently clustered into potential themes by identifying patterns of meaning across participants’ narratives. These preliminary themes were reviewed in relation to both the coded extracts and the full dataset, refined through team discussion, and interpreted in light of the Heideggerian hermeneutic phenomenological framework. This recursive process allowed the analysis to move from descriptive coding toward a more interpretative understanding of the emotional, relational, ethical, and institutional dimensions of nurses’ lived experiences.

NVivo 15 was used to support the organization and management of the qualitative data. Specifically, the software facilitated transcript storage, coding, retrieval and comparison of coded segments, analytic memo writing, thematic mapping, and documentation of analytic decisions. In this sense, NVivo 15 contributed to the transparency and auditability of the analytic process. However, the interpretation of the data remained researcher-driven and theoretically informed; the software functioned as an analytic support tool rather than as a determinant of theme development.

Reflexivity was integral to the analytic process. Analytic memos were maintained to document researchers’ interpretations, assumptions, and emotional responses, supporting critical awareness of how professional background and theoretical positioning influenced the analysis. Consistent with hermeneutic phenomenology, analysis involved iterative movement between individual data extracts and the broader experiential context, enabling an in-depth understanding of participants’ experiences.

All interviews were conducted in Spanish and transcribed verbatim. Quotations selected for inclusion in the manuscript were translated into English for publication purposes. The translated excerpts were reviewed against the original Spanish transcripts by bilingual members of the research team to ensure semantic accuracy and to preserve the emotional, contextual, and interpretative meaning of participants’ narratives. When necessary, translation choices were discussed collaboratively to achieve conceptual equivalence rather than a strictly literal translation. Formal back-translation was not performed.

### 2.7. Ethical Considerations

This study was conducted in accordance with the Declaration of Helsinki. Ethical approval was obtained from the Research Ethics Committee of the Canary Islands Health Service (reference: CHUC_2025_96).

Institutional permission was granted by the hospital management and the Head of NICU nursing. All participants received verbal and written information about the study and provided informed consent prior to participation.

Confidentiality and data protection were ensured in accordance with Spanish data protection legislation (Organic Law 3/2018) and the European General Data Protection Regulation (EU 2016/679). Participants were assigned pseudonyms to ensure anonymity.

Participation was voluntary, and participants were informed of their right to withdraw from the study at any time without consequences. No coercion was involved. All data were handled confidentially and securely throughout the research process.

### 2.8. Rigor and Reflexivity

The study adhered to the COREQ guidelines [17], ensuring coherence across the theoretical framework, methodological design, and data analysis.

Rigor was supported by transparency in the analytic process, including detailed descriptions of coding procedures, theme development, and iterative refinement. The inclusion of verbatim quotations in the results enhanced credibility by preserving participants’ voices.

The professional proximity of two interviewers to the NICU setting was considered during the reflexive process. Rather than being treated only as a limitation, this familiarity was understood as a potential source of contextual sensitivity while also requiring critical reflexive awareness. Analytic memos and team discussions were used to examine how prior clinical experience, assumptions about the unit, and shared professional language could influence data collection, interpretation, and theme development.

In addition, the research team considered the professional proximity of other members involved in the study, including prior clinical experience in the NICU and management-related familiarity with the unit. These positions were discussed reflexively during analysis to examine how insider knowledge, organizational familiarity, and shared professional language could shape interpretation.

Reflexivity was considered a central component of methodological rigor. The research team acknowledged that data interpretation was shaped by their clinical experience, professional background, and theoretical positioning. Ongoing critical reflection throughout the analytic process allowed for awareness of potential biases and assumptions.

Consistent with hermeneutic phenomenology, thematic analysis was understood as an interpretative process rather than a purely descriptive technique, enabling a deeper understanding of participants’ experiences in context. The hierarchical structure of themes and subthemes was visualized through NVivo-generated models, supporting the interpretation of relationships between experiential dimensions.

## 3. Results

Seven interconnected themes were identified through reflexive thematic analysis: emotional responses, therapeutic bond, coping strategies, perceived institutional support, training needs, infrastructure and humanization, and professional repercussions. Although analytically distinguishable, these themes are not isolated categories but rather interrelated experiential dimensions. Emotional responses emerged as a central dimension of participants’ narratives and were closely linked to therapeutic bonding, coping strategies, perceived institutional support, training needs, infrastructure and humanization, and professional repercussions. This integrated reading is introduced here to orient the presentation of the findings and is further developed in Section 4 as an interpretative synthesis.

To enhance transparency and accessibility of the qualitative findings, Table 3 summarizes the thematic structure generated through the reflexive thematic analysis. For each main theme, the table includes the corresponding subthemes, the analytical meaning attributed during interpretation, and a representative quotation illustrating how the theme was grounded in participants’ narratives. The table should be read as a synthesis of the findings, while the subsequent subsections provide a more detailed narrative account of each thematic domain.

**Figure 1:**
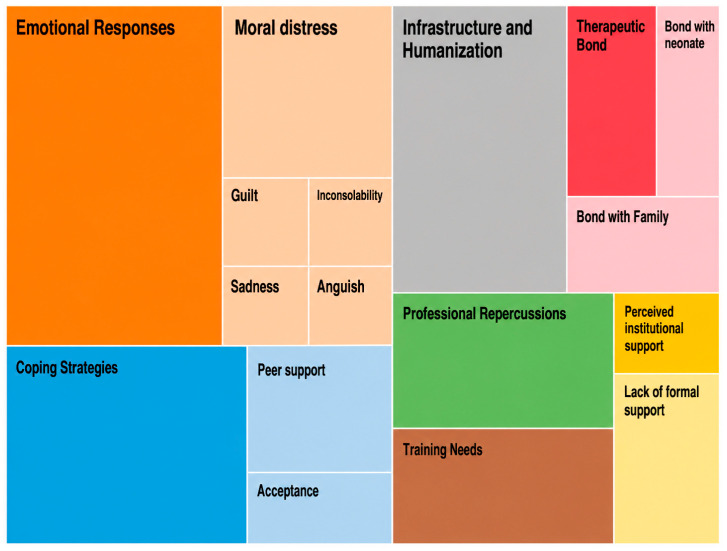
Hierarchical thematic map derived from the NVivo 15-supported analysis. The figure illustrates the hierarchical organization and relative prominence of the main themes and subthemes derived from the coding process. It is intended to enhance analytic transparency and auditability rather than to represent causal relationships between categories.

To enhance transparency in the analytic process, Figure 1 presents the hierarchical thematic structure generated from the NVivo 15-supported coding process. The figure should be interpreted as a visual representation of the organization and relative prominence of themes and subthemes, while the interpretative relationships between these dimensions are developed narratively in Section 4.

### 3.1. Emotional Responses

Emotional responses constituted the central axis of nurses’ experiences, encompassing sadness, guilt, anguish, inconsolability, and moral distress. These responses were described as intense, persistent, and context-dependent, varying according to clinical circumstances such as the duration of hospitalization and the expected prognosis.

Cases involving prolonged hospitalization or unexpected death were perceived as particularly distressing:

“In general, deaths affect me less when the time of care has been very short. However, when it comes to premature infants who have been hospitalized for months—six, seven, or eight months—and then die, the emotional impact is much greater”.

(Nurse 1)

Similarly, anticipated death was described as emotionally less disruptive, although still significant:

“It is not the same when a healthy newborn dies, with all life expectations ahead, compared to a chronic patient for whom you know that the end will come sooner or later. In those cases, although it still hurts, you feel somewhat more prepared”.

(Nurse 3)

Participants also expressed guilt and self-questioning, especially when death occurred suddenly or after intense clinical effort. Emotional responses were therefore not limited to grief alone but included moral unease, helplessness, and enduring emotional discomfort.

### 3.2. Therapeutic Bond

The therapeutic bond emerged as a key factor shaping the intensity and meaning of emotional responses. Nurses described developing deep emotional connections with both neonates and their families, particularly in prolonged admissions or complex cases.

“You create a bond. You care for them for a long time, and the loss is experienced differently. Some patients stay with you forever”.

(Nurse 1)

Another participant highlighted the relational dimension of chronic and long-term cases:

“There are some children who affect you more than others, especially those you bond with. In chronic cases, you build a relationship with both the child and the family, and that makes the impact greater”.

(Nurse 3)

Participants described this bond as extending beyond technical care and as making some losses more emotionally significant and memorable.

### 3.3. Coping Strategies

Participants reflected on several ways of coping with perinatal death, including peer support, emotional distancing, acceptance, and relational presence.

Peer support emerged as a major protective resource:

“There are very experienced colleagues in the unit. I have always felt supported by them. Sometimes, just making a phone call—even on a weekend—was enough to receive peer support”.

(Nurse 5)

Emotional distancing was also described as a necessary mechanism to remain functional:

“One of the key aspects is the ability to disconnect. It’s a survival mechanism—otherwise, it would be impossible to work in this profession”

(Nurse 4)

At the same time, some participants described a more reflective and relational way of coping, based on presence and accompaniment:

“You still feel, you still suffer, but you learn to accompany from another place. Not by trying to encourage or through empty silence, but through presence—listening, respecting timing, being available. A real and human form of support”.

(Nurse 1)

Overall, participants described coping strategies as ways of managing emotional burden while continuing to provide care in the NICU.

### 3.4. Perceived Institutional Support

Participants consistently reported significant limitations in institutional support. While informal support among colleagues was highly valued, formal systems were often perceived as insufficient or absent.

“As professionals, we need more resources, more staff, and more institutional support. End-of-life care is not limited to a shift—it requires continuous presence, and that demands proper organizational structure and support”.

(Nurse 2)

Another participant explicitly pointed to the lack of formal psychological support:

“There is no formal professional support. There is no psychologist available for staff, and that seems completely insufficient. Talking to colleagues helps, but there is no structured support system to process these experiences”.

(Nurse 3)

Participants therefore expressed a contrast between the emotional demands of perinatal loss and the limited formal support available within the unit.

### 3.5. Training Needs

Training needs emerged as a distinct and recurrent theme. Participants emphasized the need for ongoing, targeted education in neonatal end-of-life care, especially in communication, bereavement support, and emotional management.

“It would be important to have regular training updates, as well as spaces for emotional and professional reflection”.

(Nurse 1)

Another participant reinforced the importance of specific training:

“Training is essential—continuous training, workshops on perinatal bereavement, and end-of-life care in neonatal and pediatric patients. Support groups are also important”.

(Nurse 3)

Training was described not only as a means of improving technical competence but also as contributing to broader professional education, strengthening emotional preparedness and confidence in accompanying families through loss.

### 3.6. Infrastructure and Humanization

Participants identified significant limitations in both the physical infrastructure and organizational conditions, which adversely affected the quality of care during perinatal death. Lack of privacy, inadequate spaces, and environmental exposure were described as major barriers to humanized care.

“End-of-life is not ‘normal’ for a family, and it must be treated with dignity and care. Everything should be done properly and in a coordinated way, because accompanying is also caring”.

(Nurse 1)

The absence of private spaces for professionals was also highlighted:

“Professionals also need spaces. It is not normal that the only option is to go and cry in a bathroom. That says a lot about how the system is organized”.

(Nurse 5)

Although some participants acknowledged improvements in recent years, they also stressed that significant gaps remain:

“There have been positive changes, especially in terms of humanization, but something fundamental is still missing—privacy and adequate physical spaces”.

(Nurse 10)

Participants associated humanized end-of-life care not only with interpersonal attitudes but also with privacy, adequate spaces, and organizational conditions.

### 3.7. Professional Repercussions

Participants’ accounts connected emotional responses, therapeutic relationships, and perceived institutional limitations with professional and personal repercussions. Participants shared emotional exhaustion, self-doubt, compassion fatigue, and persistent rumination.

“Sometimes you feel that maybe you could have done more for that child, even though you know rationally that it is not always the case”.

(Nurse 1)

Similarly:

“I often feel that I could have done better. I don’t know if it’s due to lack of preparation, resources, or experience, but it’s a frequent feeling”.

(Nurse 8)

The emotional burden was not confined to the workplace:

“You go home feeling affected. You keep thinking about it, it stays with you for hours. Over time, it settles, but it does not disappear”.

(Nurse 9)

These repercussions were described as cumulative and, in some cases, as influencing how nurses experienced their professional role, their self-confidence, and their capacity to continue working in highly demanding environments.

## 4. Discussion

This study provides a phenomenological understanding of NICU nurses’ lived experiences of perinatal death as a multidimensional phenomenon involving emotional, relational, ethical, and organizational dimensions of care.

Rather than discrete categories, the findings suggest an integrated experiential structure in which emotional responses emerged as a central dimension, closely linked to therapeutic bonding, coping strategies, perceived institutional support, training needs, infrastructure, and professional repercussions. These relationships should be understood as interpretative connections derived from participants’ narratives, rather than as causal or longitudinal associations.

### 4.1. Emotional Responses Within an Ethical and Clinical Context

The findings suggest that perinatal death is experienced as an intense and persistent emotional event characterized by sadness, guilt, anguish, and moral distress. These results are consistent with previous research describing grief among neonatal nurses as cumulative and often unrecognized [13], as well as with evidence linking repeated exposure to neonatal death with burnout and post-traumatic stress symptoms [8]. They also align with studies highlighting the emotional and relational complexity inherent in neonatal nursing care [20]. Beyond individual emotional burden, these experiences are also shaped by broader sociocultural dynamics. Perinatal death has been described as a form of disenfranchised grief, often lacking social recognition and shared mourning practices. This invisibility may contribute to emotional isolation among both families and healthcare professionals, intensifying the complexity of neonatal end-of-life care [21].

However, the present findings extend this understanding by situating emotional responses within an ethical and clinical framework. Participants frequently interpreted death as a form of professional failure, particularly in highly technological environments oriented toward survival. This perception reflects the concept of moral distress [10], especially in situations involving prolonged life-sustaining treatments despite poor prognosis, reinforcing ethical tensions described in NICU settings [11]. These tensions have also been conceptualized as bioethical challenges related to proportionality of care, uncertainty, and shared decision-making with families [22,23].

Although emotional suffering, ethical uncertainty, and professional self-doubt were closely interrelated in participants’ accounts, they were analytically distinguished in the interpretation. Emotional suffering referred mainly to grief, sadness, anguish, helplessness, and persistent emotional discomfort associated with neonatal loss. Ethical uncertainty and moral distress referred to situations in which nurses perceived tension between clinical decisions, prognostic uncertainty, technological possibilities, family needs, and their own professional values. Professional self-doubt referred to participants’ questioning of their own actions, preparedness, or perceived capacity to provide adequate care. Differentiating these dimensions helped clarify that moral distress was not treated as equivalent to all forms of emotional suffering but as a specific ethical component within a broader emotional and professional experience.

### 4.2. Relational Proximity and the Therapeutic Bond

A key contribution of this study is the identification of the therapeutic bond as an important relational dimension that appeared to intensify the meaning of loss.

This finding is consistent with qualitative evidence suggesting that relational proximity is a key determinant of emotional burden among NICU professionals [24]. From a phenomenological perspective, this bond represents a form of embodied and relational engagement that transcends technical care [25,26], positioning nurses not only as care providers but as participants in the family’s lived experience of loss.

At the same time, this relational closeness increases vulnerability, as emotional involvement intensifies the impact of death and complicates processes of emotional detachment. In this context, limited preparation in supporting grieving families may further exacerbate emotional strain [27].

### 4.3. Coping Strategies for Managing Emotional Burden

The coping strategies identified in this study reflect a continuous effort to balance emotional engagement with professional sustainability. Mechanisms such as peer support, emotional distancing, and meaning-making function as regulatory processes that enable nurses to sustain their practice in emotionally demanding environments.

These findings resonate with the concept of vicarious resilience [2], whereby professionals derive meaning and strength from accompanying families through loss. In addition, reflective practice and self-compassion have been identified as key components in sustaining compassionate care [14].

Nevertheless, the findings also highlight that these strategies operate primarily at an individual level and may be insufficient in the absence of structured organizational support.

### 4.4. Institutional and Structural Conditions in the Experience of Perinatal Death

The findings suggest that institutional and structural conditions played an important contextual role in shaping participants’ experiences and management of the emotional burden of perinatal death. Nurses consistently reported limited access to formal psychological support, structured debriefing, and organizational frameworks specifically designed to address the emotional impact of neonatal end-of-life care.

These findings align with previous research identifying gaps in institutional responses to neonatal end-of-life care, particularly in Ibero-American contexts [12]. In the present study, participants revealed a contrast between the emotional demands of perinatal loss and the limited formal resources available to support professionals. This suggests that, although important, individual coping strategies alone may be insufficient without structured institutional support.

This institutional dimension was further reinforced by training needs. Participants emphasized the importance of education in communication, bereavement care, neonatal palliative care, and ethical decision-making, in line with existing literature [28]. Training was therefore conceptualized not only as a technical or educational resource but also as a contributor to emotional preparedness and professional confidence in the provision of end-of-life care for families.

Infrastructural limitations were also identified as relevant barriers to humanized care. Lack of privacy, inadequate spaces for families, and limited areas for professionals to process emotional experiences were described as factors that could make end-of-life care more difficult. From this perspective, humanization extends beyond interpersonal interaction and also depends on material, spatial, and organizational conditions that support dignity, privacy, and accompaniment.

The relationship between infrastructure and professional repercussions was particularly relevant in participants’ accounts. Limited privacy, lack of dedicated spaces for families, and the absence of areas where professionals could pause or process emotionally difficult situations were described as barriers not only to family-centered care but also to nurses’ emotional containment. Within this context, the physical environment may contribute to the persistence of emotional discomfort, rumination, and perceived difficulty in recovering after highly distressing events. These findings suggest that infrastructure should be understood not merely as a logistical issue but as part of the emotional and ethical ecology of neonatal end-of-life care.

Spiritual and existential dimensions of neonatal care have also been recognized as relevant components of humanized practices [29,30]. In this context, palliative approaches oriented towards a peaceful and dignified end of life remain central [31,32]. However, in this study, these implications should be understood as context-specific and derived from participants’ accounts within a single tertiary-level NICU. Overall, institutional support, training, infrastructure, and humanization were interpreted as interconnected contextual conditions that shaped how nurses described their ability to provide care, accompany families, and process perinatal loss.

### 4.5. Professional Repercussions and Professional Self-Questioning

The interaction between emotional responses, relational proximity, and institutional conditions was associated with relevant professional repercussions in participants’ accounts. Participants described emotional exhaustion, self-doubt, and compassion fatigue, consistent with evidence linking repeated exposure to patient death to adverse psychological outcomes [1,4].

Rather than indicating a uniform transformation of professional identity, the findings suggest that perinatal death prompted processes of professional self-questioning and reflection on the meaning and limits of care. Some nurses reported feelings of guilt or perceived failure, particularly when death occurred after prolonged care or intense clinical effort. These experiences appeared to affect how participants understood their professional role and their capacity to continue working in emotionally demanding environments.

These findings align with qualitative research describing professional grief as a cumulative process that may influence professional trajectories over time [33,34]. Furthermore, the coexistence of distress and post-traumatic growth highlights the complexity of adaptive responses among professionals exposed to repeated loss [35].

Importantly, the present findings suggest that the repercussions of perinatal death may extend beyond the clinical encounter, affecting both personal well-being and professional self-understanding.

### 4.6. Towards an Integrated Interpretative Model

Taken together, the findings suggest an integrated interpretative model in which emotional responses were not experienced as isolated individual reactions but as situated experiences embedded in relational, ethical, and organizational contexts.

Within this model, therapeutic bonding appeared to intensify the emotional meaning of perinatal loss, while coping strategies were described by participants as ways of managing emotional burden and maintaining professional continuity. Perceived institutional support, training needs, and infrastructural conditions were not interpreted as causal determinants but as contextual factors that participants associated with their ability to process loss, accompany families, and sustain emotionally demanding care.

Therefore, the integrated model should be understood as a conceptual synthesis of the relationships suggested by participants’ narratives. It highlights how emotional responses, relational proximity, coping resources, and perceived organizational conditions were interconnected in nurses’ accounts of perinatal death, while avoiding causal assumptions beyond the scope of the qualitative data.

### 4.7. Strengths and Limitations of This Study

This study has several strengths. The use of a hermeneutic phenomenological approach enabled an in-depth exploration of the meanings that NICU nurses attributed to perinatal death, moving beyond predominantly quantitative approaches focused on burnout, compassion fatigue, or psychological symptoms. The richness of participants’ narratives provided insight into emotional, relational, ethical, and institutional dimensions that are often difficult to capture through quantitative approaches alone. In addition, the relational and multidimensional interpretation of the findings illustrates how emotional responses, therapeutic bonding, coping strategies, perceived institutional support, and professional repercussions appeared interconnected in participants’ accounts.

Methodologically, the coherence between the Heideggerian hermeneutic phenomenological framework, reflexive thematic analysis, and reflexivity procedures strengthened the credibility and interpretative depth of the study. The inclusion of nurses with different levels of NICU experience, parental status, gender, and previous training in palliative, bereavement, or end-of-life care enriched the thematic analysis by allowing the phenomenon to be explored from different professional and personal positions within the same clinical context. In addition, analytic memos, collaborative team discussions, and the NVivo 15-supported audit trail contributed to transparency and auditability.

Limitations should also be acknowledged. First, the study was conducted in a single tertiary-level NICU in Spain; therefore, participants’ experiences may have been shaped by the organizational culture, staffing conditions, emotional support systems, bereavement care practices, and neonatal care routines of this particular institution. The findings should consequently be understood as context-dependent and potentially transferable to similar NICU environments, rather than generalizable to all neonatal care settings or healthcare systems.

Second, although the sample size was appropriate for a hermeneutic phenomenological study, the inclusion of ten nurses may have limited the range of perspectives captured. Nurses with different coping styles, levels of experience, or degrees of exposure to perinatal death may have provided additional nuances. Moreover, self-selection bias cannot be excluded, as nurses with stronger emotional experiences, greater interest in bereavement care, or greater willingness to discuss sensitive situations may have been more likely to participate.

Third, the retrospective and self-reported nature of the interviews may have influenced participants’ narratives through memory reconstruction, subsequent emotional processing, or later clinical experiences. Data were also collected exclusively through interviews, without triangulation through observational data, reflective diaries, field notes, or institutional documentation, which may have limited the contextual depth and confirmability of the findings.

Finally, the study focused exclusively on nurses’ perspectives. The absence of interdisciplinary viewpoints, including those of physicians, psychologists, social workers, or parents, limits understanding of how perinatal death is experienced across different professional, family, and relational contexts within NICU care. Interviews were conducted within the workplace setting, which may have contributed to social desirability bias when discussing institutional limitations or emotional vulnerability.

Although reflexivity was addressed throughout data collection and analysis, the researchers’ professional backgrounds, prior clinical experience, and familiarity with neonatal care may have influenced interpretation. In addition, because interviews were conducted in Spanish and selected quotations were translated into English, some emotional nuances, culturally embedded meanings, or idiomatic expressions may not have been fully preserved, although translations were reviewed by bilingual members of the research team.

### 4.8. Recommendations for Further Research

The alignment between the results and discussion highlights that nurses’ emotional responses to perinatal death are shaped by relational proximity, ethical uncertainty, institutional culture, and level of preparedness. The training gaps identified in this study are consistent with previous research highlighting the need for structured training in end-of-life communication and neonatal palliative care [28,36].

Future research should focus on evaluating the effectiveness of institutional interventions aimed at supporting healthcare professionals, including structured debriefing sessions, accessible psychological support, and simulation-based training in end-of-life care. In addition, further studies are needed to explore how NICU environments can be redesigned to facilitate privacy, emotional expression, and humanized care.

Longitudinal research could provide valuable insight into the cumulative impact of repeated exposure to perinatal death on nurses’ well-being and professional trajectories, particularly in relation to burnout and compassion fatigue, as described in recent pediatric nursing literature [1,4,8].

### 4.9. Context-Specific Relevance for Clinical Practice

The findings of this study may inform context-specific implications for NICU settings with similar organizational and cultural characteristics. Within the studied context, participants identified the relevance of greater institutional attention to professionals’ emotional well-being, particularly through training in neonatal palliative care, end-of-life communication, bereavement support, reflective spaces, and access to psychological support.

Participants’ accounts also suggest that improvements in privacy, family spaces, and professional self-care environments may support more humane and family-centered end-of-life care. These implications should be interpreted cautiously, as they derive from a single tertiary-level NICU and may need to be adapted to the organizational culture, staffing conditions, resources, and bereavement care practices of each clinical setting.

Giving voice to NICU nurses’ experiences is particularly relevant in Spanish and Ibero-American neonatal care contexts, where previous research has identified the need for more structured training, institutional support, and bereavement care protocols. Participants’ narratives make visible the emotional work, professional grief, ethical uncertainty, and perceived lack of formal support that may remain underrecognized in highly technological neonatal care environments.

In this regard, adopting a biopsychosocial model of care may provide a useful framework for addressing the complex interplay between emotional, relational, family, and organizational factors in NICU settings [37]. Such an approach may support more compassionate, holistic, and sustainable neonatal care, while acknowledging that specific interventions should be adapted to each institutional context.

From a practical perspective, the findings may support the development of scalable, context-sensitive interventions in NICU settings. These could include structured post-event debriefing after neonatal deaths, peer-support mechanisms facilitated by experienced nurses, access to psychological support for staff, simulation-based training in end-of-life communication, and unit-level bereavement care protocols. In addition, environmental improvements such as private family rooms, spaces for farewell rituals, and designated areas for professionals’ emotional decompression may help support more humane care. These strategies should be adapted to the resources, staffing conditions, organizational culture, and bereavement care practices of each institution.

## 5. Conclusions

Perinatal death in Neonatal Intensive Care Units was experienced by participants as a profound emotional, ethical, relational, and professional challenge. The findings suggest that nurses’ emotional responses should not be understood as isolated individual reactions but as situated experiences emerging within prolonged relationships with neonates and families, ethical uncertainty, technological intensity, and perceived institutional conditions.

Participants described sadness, guilt, moral distress, persistent memories, and professional self-questioning, particularly in situations involving prolonged hospitalization, unexpected death, or limited institutional support. Therapeutic relationships and accompaniment were also described as meaningful dimensions of care, while peer support emerged as an important coping resource.

Given the single-site design of this phenomenological study, these findings should be understood as context-dependent and potentially transferable to similar NICU environments rather than generalizable to all neonatal care settings. Within this context, the study highlights the importance of strengthening professional support through targeted training, reflective spaces, accessible psychological support, and improvements in privacy, humanization, and infrastructure. Integrating attention to nurses’ emotional well-being may contribute to more compassionate, ethical, and sustainable neonatal end-of-life care.

## Figures and Tables

**Table 1:** Sociodemographic and professional characteristics of participants.

Participant	Gender	Age	Parenthood Status	Years in NICU	Previous Training in Palliative, Bereavement, or End-of-Life Care
Nurse 1	Female	52	Yes	28	Yes
Nurse 2	Female	39	Yes	18	Yes
Nurse 3	Female	47	Yes	25	Yes
Nurse 4	Female	32	No	6	No
Nurse 5	Female	32	No	6	No
Nurse 6	Male	29	No	6	Yes
Nurse 7	Female	51	Yes	24	Yes
Nurse 8	Female	28	No	6	Yes
Nurse 9	Male	31	No	5	Yes
Nurse 10	Male	35	No	5	Yes

Note: NICU = Neonatal Intensive Care Unit. All participants had previous clinical experience caring for neonates and families in situations of perinatal death.

**Table 2:** Semi-structured interview guide organized by conceptual domains.

Conceptual Domain	Interview Questions/Prompts
Meaning of neonatal end-of-life care	What does end-of-life care for your patients mean to you?
First experiences and emotional responses	Do you remember how you felt when your first patient died in the NICU? What were your reactions?
Preparedness and coping	Do you feel prepared to face the possibility of a neonatal death? How do you cope with it?
Family communication and bereavement support	Do you feel prepared to support families during perinatal death? Do you think your communication with families in bereavement situations could improve? How difficult has it been to support parents when a patient has died or may die?
Technology, care environment, and humanization	Do you think technology in NICUs sometimes limits communication with families? Are there adequate spaces for families experiencing perinatal loss?
Professional impact and support needs	Have you ever felt the need to leave the unit due to a perinatal death? Are there support systems for professionals dealing with emotional burden?
Training and improvement proposals	Do you think more training is needed in neonatal end-of-life care? What changes or resources would help improve care for families and professionals?

Note: The semi-structured interview guide was used flexibly. Follow-up prompts were introduced when necessary to deepen participants’ narratives and clarify meanings, while preserving the participant-led nature of the interview.

**Table 3:** Main themes, subthemes, analytical meanings, and representative quotations.

Main Theme	Subthemes	Analytical Meaning	Representative Quotation
**Emotional responses**	Guilt; sadness; anguish; inconsolability; moral distress	Perinatal death was experienced as an intense emotional event involving grief, helplessness, self-questioning, and ethical discomfort, particularly in prolonged or unexpected deaths.	*“When it comes to premature infants who have been hospitalized for months—six, seven, or eight months—and then die, the emotional impact is much greater” (Nurse 1).*
**Therapeutic bond**	Bond with neonate; bond with family	Prolonged care created strong emotional connections with neonates and families, intensifying the meaning and impact of loss.	*“You create a bond. You care for them for a long time, and the loss is experienced differently” (Nurse 1).*
**Coping strategies**	Peer support; emotional distancing; acceptance	Nurses described coping as a process of balancing emotional engagement with professional sustainability through peer support, emotional regulation, and acceptance.	*“Sometimes, just making a phone call—even on a weekend—was enough to receive peer support” (Nurse 5).*
**Perceived institutional support**	Lack of formal support	Informal support among colleagues was valued, but formal psychological and organizational support was perceived as insufficient.	*“There is no formal professional support. There is no psychologist available for staff” (Nurse 3).*
**Training needs**	—	Participants identified the need for continuous training in neonatal end-of-life care, bereavement support, communication, and emotional management.	*“Training is essential—continuous training, workshops on perinatal bereavement, and end-of-life care in neonatal and pediatric patients” (Nurse 3).*
**Infrastructure and humanization**	—	Physical and organizational conditions, including lack of privacy and dedicated spaces, were perceived as barriers to humanized end-of-life care.	*“It is not normal that the only option is to go and cry in a bathroom” (Nurse 5).*
**Professional repercussions**	—	Emotional burden extended beyond the clinical encounter, affecting nurses’ professional confidence, well-being, and perception of their role.	*“You go home feeling affected. You keep thinking about it, it stays with you for hours” (Nurse 9).*

Note: Quotations were selected to illustrate the central meaning of each theme. Themes without subthemes were retained as coherent experiential domains.

## Data Availability

The data supporting the findings of this study are available from the corresponding author upon reasonable request. The data are not publicly available due to privacy and ethical restrictions.

## Abbreviations

The following abbreviations are used in this manuscript:

NICU	Neonatal Intensive Care Unit
COREQ	Consolidated Criteria for Reporting Qualitative Research
